# The key role of cognitive fusion linking mindfulness and personality: a cross-sectional study

**DOI:** 10.1038/s41598-025-18324-z

**Published:** 2025-09-23

**Authors:** Daniel Heppt, Niklas Bergmann, Ingmar Conell, Inge Hahne, Eric Hahn, Thi Minh Tam Ta, Kerem Böge

**Affiliations:** 1 Department of Psychiatry and Neuroscience, Charité – Universitätsmedizin Berlin, Campus Benjamin Franklin, Berlin, Germany; 2 Department of Psychology, Medical University Brandenburg, Neuruppin, Germany; 3 Deutsches Zentrum Für Psychische Gesundheit (DZPG), Berlin, Germany

**Keywords:** Mindfulness, Meditation, Personality traits, Big five model of personality, Cognitive fusion, Psychological flexibility, Neuroscience, Psychology

## Abstract

The present article explores the relationship between mindfulness meditation and personality. Based on a substantial body of research, mindfulness has been shown to have significant associations with personality. Recent research suggests that mindfulness meditation may influence personality traits in accordance with the Five Factor Personality Model. To examine this, we investigated whether mindfulness mediates the relationship between meditation experience and personality in an international, cross-sectional sample of N = 779 meditators (mean meditation experience = 6.4 years, 69.8% female). Additionally, the mechanism of how mindfulness and personality are associated remains largely unclear. We therefore assessed whether cognitive fusion, a measure of psychological flexibility, functions as a mediator between both constructs. Our analyses indicate that mindfulness serves as a significant mediator for all five personality traits, supporting the hypothesis that mindfulness meditation may contribute to changes in personality traits, particularly by reducing neuroticism. As the mediated effect was strongest for neuroticism, this underscores the potential of mindfulness meditation to possibly have an influence on mental wellbeing. Cognitive fusion significantly mediated the effect of mindfulness on neuroticism (β = − 0.40, 95% CI [–.45, –.34]) and agreeableness (β = 0.17, 95% CI [0.10, 0.24]), while fully mediating the association with conscientiousness (β = 0.17, 95% CI [0.10, 0.25]). These results suggest that enhanced cognitive flexibility accounts for a significant proportion of mindfulness’s impact on personality. Our results further revealed a previously unidentified pathway wherein cognitive fusion fully mediated the relationship between mindfulness and extraversion (β = 0.19, 95% CI [0.11, 0.27]), a trait typically displaying a weak association with mindfulness. These findings suggest cognitive fusion’s central role in linking mindfulness and personality and offer a promising foundation for future explorations in this field.

## Introduction

Mindfulness, as defined by Kabat-Zinn, involves deliberate engagement with the present moment, emphasizing openness, acceptance, and nonjudgment^1^. While rooted in Eastern spiritual traditions, it has become a focal point of psychological research and interventions. Scientific interest has surged in recent decades, with over 16,000 publications on mindfulness since 1966^2^. This substantial body of research has documented beneficial effects of mindfulness on mental and physical well-being^3^. Specifically, previous findings highlight the potential of mindfulness meditation (MM) in decreasing anxiety and depressive symptoms^4^, promoting self-compassion and life-satisfaction^5,6^, and reducing chronic pain intensity^7^. The implications of these findings extend beyond theoretical explorations to applied interventions, including mindfulness-based stress reduction (MBSR)^8^ and mindfulness-based cognitive therapy (MBCT)^9^.

Amid this evolving landscape, researchers aim to explore the relationship between mindfulness and personality^10^. Commonly described with the Big Five model, personality encompasses five traits—neuroticism, extraversion, openness to experience, agreeableness and conscientiousness. These traits represent a constellation of persistent cognitive, affective, and behavioral patterns that distinguish individuals and shape their engagement with their environment^11^. The meta-analyses by Hanley and Garland^10^ and Giluk^12^ comprehensively summarize the literature on mindfulness and personality. Both meta-analyses highlight the strongest correlation between mindfulness and neuroticism, with *r* = − 0.45 and *r* = − 0.47 respectively. Neuroticism is a personality trait characterized by heightened emotional reactivity, a tendency towards negative emotions such as anxiety and depression, and difficulty coping with stressors effectively^13^. This negative correlation implies that higher levels of mindfulness are linked to lower levels of neuroticism.

Conversely, all other personality traits exhibit positive correlations (conscientiousness *r* = 0.32 and *r* = 0.34; agreeableness *r* = 0.22 and *r* = 0.26; openness *r* = 0.15 and* r* = 0.15; extraversion *r* = 0.12 and* r* = 0.17), suggesting that increased mindfulness scores are associated with higher scores in those traits. Specifically, conscientiousness and agreeableness emerge as the next most strongly correlated traits. Conscientiousness is characterized by organization, responsibility, and goal-directed behavior, while agreeableness reflects a tendency towards kindness, cooperation, and empathy in interpersonal relationships^13^. Extraversion, characterized by sociability and assertiveness, and openness to experience, defined by curiosity and a willingness to embrace new experiences, demonstrates the least pronounced correlations with mindfulness^13^.

Research in this field is especially relevant since people vary in their dispositions towards mindfulness practices, resulting in diverse outcomes from such engagements^14^. By investigating these differences in personality traits, researchers may be able to forecast which individuals are likely to benefit from specific types of mindfulness approaches, paving the way for tailored psychological treatments and a more personalized approach to mindfulness-based interventions. In this regard, de Vibe et al. observed that medical and psychology students with high baseline scores in neuroticism who participated in a mindfulness-based stress reduction course displayed more pronounced reductions in mental distress^15^. Conversely, the influence of mindfulness practice on personality structure remains more elusive.

Exploring this research question, a randomized controlled trial was conducted with a sample of Norwegian medical and psychology students (*N* = 288)^16^. While the effect size of mindfulness training on neuroticism was small (Cohen’s *d* = 0.28), their findings demonstrated that the reduction in neuroticism following a seven-week MBSR course persisted over a six-year follow-up period. This suggests that MM may have a lasting impact on personality. By potentially influencing personality structure, particularly by decreasing neuroticism, meditative practice may reduce the likelihood of experiencing anxiety, depression and related symptoms^17^, offering lasting impacts on psychological well-being^18,19^. Historically, personality traits have been perceived as consistent, displaying only slight change throughout adulthood^20^. More recent research, however, showed that personality can be subject to change^21^. According to Buddhist teachings, MM can profoundly alter the sense of self^22^. In line with this, Hölzel et al. suggest that mindfulness can reduce attachment to a fixed self-concept, promoting greater flexibility in how the self is experienced^23^.

Nyklíček et al.^24^ and the literature review of Crescentini and Capurso^25^, further support the notion that MM may be able to affect personality structure by suggesting that mindfulness meditation may shape personality towards more healthy profiles. However, the studies compared by Crescentini and Capurso are limited by small sample sizes and inconsistencies with major meta-analyses on the correlation between mindfulness and personality. Additionally, relatively few studies have examined this research question, likely due to the methodological difficulties associated with such investigations. These issues emphasize the necessity for additional research. To further investigate this topic, we propose and test a mediation framework in which mindfulness functions as a mediator between meditation experience and personality traits. This hypothesis aligns with evidence showing that mindfulness increases with greater meditation experience^26^, and that, as previously stated, higher mindfulness is associated with lower neuroticism and higher levels of conscientiousness, agreeableness, extraversion, and openness^10^.

Beyond the influence of meditation experience on personality structure, another area of interest is how mindfulness affects personality. Mindfulness shares considerable overlap with several psychological constructs. One of these, cognitive fusion, is a core component of psychological inflexibility and refers to the tendency of overly identifying or “fusing” with one’s thoughts. When those thoughts are negative, this can lead to increased emotional distress^27^. This phenomenon involves getting caught up in the content of thoughts, rather than recognizing them as transient mental events, ultimately constraining one’s adaptability^28^.

Cognitive fusion shows a strong negative correlation with mindfulness (*r* =− 0.71)^27^, indicating that higher levels of mindfulness are linked to lower levels of cognitive fusion. This association seems intuitive since core elements of MM are observation, non-judgment and acceptance of one’s thoughts. Although mindfulness shares similarities with cognitive fusion, both constructs have been shown to exist independently^27^. Recent studies have investigated the role of cognitive fusion in the context of mindfulness. Pux et al.^29^ reported that cognitive fusion mediates the relationship between mindfulness and negative affect. Likewise, Nitzan-Assayag et al.^30^ demonstrated that cognitive fusion also mediates the effects of mindfulness on depression and post-traumatic stress symptoms. These results underscore the importance of considering cognitive fusion as a mediator in mindfulness-related effects. Additionally, personality has been linked to cognitive fusion within the broader context of psychological inflexibility, as all personality traits except extraversion have been shown to predict psychological inflexibility^31^. Thus, cognitive fusion has been shown to have significant associations with mindfulness and personality traits, providing a strong rationale for exploring its mediating role.

Against this background, the present study will examine two hypotheses. First, we aim to investigate the potential transformative effects of mindfulness meditation on personality structure. We anticipate that mindfulness mediates the relationship between meditation experience and personality traits. As a second hypothesis, we will investigate how mindfulness affects personality traits and expect cognitive fusion to mediate the association between both these two constructs. To account for potential confounding influences, we included two covariates in both models. The first one is meditation intensity, defined as the number of hours of meditative practice per week. The inclusion of this variable is necessary, as the amount of time spent meditating per week significantly predicts mindfulness levels^26^. Age functions as the second covariate, since older individuals tend to report higher scores in mindfulness^32^, agreeableness and conscientiousness^33^. In contrast, neuroticism tends to decline from adolescence to adulthood^33^.

## Method

### Procedure & participants

The data for this study are based on an international cross-sectional online survey, conducted between June 2020 and August 2021. In total, 950 regular meditators from the general population completed questionnaires about several psychological constructs, including mindfulness, cognitive fusion and personality traits. Participants also provided sociodemographic and meditation-related data, as well as a history on psychopathology.

The inclusion criteria for participation in this voluntary and uncompensated study required participants to be 18 years or older and have proficiency in the English language. To be included as a regular “meditator”, participants had to report at least 1 month of meditation experience. Several studies on mindfulness chose the same cut-off^29,34^, ensuring sufficient mindfulness skills while fostering diversity within the meditator sample. Another argument for setting the inclusion criteria to one month of meditation experience is that the changeability of personality traits is part of the first hypothesis and personality traits have been shown to change after the first month of treatment intervention^35^.

Participants were excluded if they reported less than one month of meditation experience, were under 18 years of age, reported implausible age values, had an age incompatible with their meditation experience or had missing data in the questionnaires.

Ethical approval (Reference: EA4/127/20) was obtained from the ethics committee of Charité–Universitätsmedizin Berlin. Recruitment leveraged online platforms dedicated to meditation, including teacher registries and meditation communities. The study, facilitated through the Unipark Software Questback platform, took approximately 20 min to complete.

### Measures

This study evaluated the following three psychological constructs with self-report questionnaires: Mindfulness, Personality traits according to the Big-Five model, and Cognitive Fusion.

#### Mindfulness

The evaluation of mindfulness involved applying the Southampton Mindfulness Questionnaire (SMQ). The questionnaire comprises 16 items, and participants rate their responses on a 7-point Likert scale (0 = strongly disagree, 6 = strongly agree). The SMQ assesses the extent of mindful responses to distressing cognitions, focusing on state mindfulness. It has been widely used in both clinical and non-clinical English-speaking samples and demonstrates robust psychometric properties (α = 0.89)^36,37^. In this sample, Cronbach’s α was 0.92.

#### Personality according to the big five inventory

The study explored personality traits, specifically neuroticism, conscientiousness and agreeableness utilizing the Big Five Inventory (BFI)^38^. This inventory comprises 44 items, rated on a 5-point Likert scale (1 = disagree strongly, 5 = agree strongly), assessing the subdomains of the Five Factor Personality Model. The BFI has been extensively validated across diverse populations and research contexts, reporting strong internal consistency values (α = 0.83)^39^. In this study, Cronbach’s α was 0.90 overall, with subscale values of 0.84 for Extraversion, 0.76 for Agreeableness, 0.81 for Conscientiousness, 0.86 for Neuroticism, and 0.76 for Openness.

#### Cognitive fusion

Assessment of cognitive fusion utilized the Cognitive Fusion Questionnaire (CFQ)^27^, featuring seven items rated on a 7-point Likert scale (1 = never true, 7 = always true). Elevated scores indicate greater fusion with inner experiences and decreased cognitive flexibility. The CFQ has been widely applied in both clinical and non-clinical English-speaking populations and exhibits robust psychometric properties, with high internal reliability (α = 0.90)^27,40^. Cronbach’s α for this sample was 0.95.

## Data analysis

All analyses were conducted using R Software (version 4.2.2). The mediation analysis was performed with the PROCESS package^41^. Sample size determination followed Fritz & MacKinnon’s simulation method^42^. A minimum of 558 participants were determined for detecting small effects on paths a and b with a power of 0.8 in a mediation analysis using percentile bootstrapping.

Correlation analyses were conducted to confirm significant relationships between mindfulness, the mediators, and personality traits. Linearity and normality of residuals was assessed through visual inspections of plots. The Breusch-Pagan test evaluated assumptions of homoscedasticity and multicollinearity was tested using the Variance Inflation Factor (VIF). Bootstrapping with 10,000 repetitions was applied to address any deviations from the normality of residuals or homoscedasticity. PROCESS model 4 was used to conduct both mediation analyses^41^. The results are presented mainly for the three most relevant personality traits associated with mindfulness—neuroticism, conscientiousness and agreeableness.

To test the first hypothesis, specifically exploring the impact of mindfulness practice on personality traits, mindfulness serves as the mediator between the independent variable (IV) meditation experience and the dependent variable (DV) personality traits. Meditation experience was quantified in months of experience, and restricted to values ≥ 1 month, while the upper cut-off was set to 800 months (approximately 67 years), as that included the oldest meditator with the longest realistic meditation experience. The second mediation analysis investigated cognitive fusion as the mediator, with mindfulness and personality serving as independent and dependent variable respectively.

## Results

Of the 950 individuals who completed the questionnaire, 171 responses were excluded from our analysis. Sociodemographic and meditation related data of participants can be found in Table 1. Correlations between variables are displayed in Table 2.

**Table 1:** Sociodemographic and meditation-related information.

Measures	Percentage/Value
Age in years—mean (SD)	39.8 (16.4)
Gender	
Female	69.8%
Male	28.7%
Diverse	1.5%
Region of residency	
Europe	52.5%
North America	30.6%
Australia and New Zealand	8.5%
Asia	6.9%
South America	0.9%
Africa	0.6%
Amount of Meditation	
Weekly Meditation Hours—mean (SD)	4.8 (13.2)
Meditation Experience in Months—mean (SD)	76.7 (118.1)

**Table 2:** Correlations between variables.

Variables	1	2	3	4	5	6	7	8	9	10
1. Age	–									
2. Gender	0.05	–								
3. SMQ	0.38***	0.10	–							
4. CFQ	−0 .37***	− .0.05	− 0.75***	–						
5. Medexp	0.54***	0.14**	0.35***	− 0.33***	–					
6. BFI_N	− 0.31***	− 0.10*	− 0.65***	0.73***	−0 .29***	–				
7. BFI_C	0.26***	− .0.14**	0.32***	− 0.36***	0.23***	− 0.37***	–			
8. BFI_A	0.28***	− 0.09	0.42***	− 0.43***	0.21***	− 0.48***	0.42***	–		
9. BFI_O	0.29***	0.04	0.32***	− 0.27***	0.26***	− 0.32***	0.28***	0.38***	–	
10. BFI_E	0.09	− 0.07	0.21***	− 0.27***	0.06	− 0.34***	0.26***	0.29***	0.31***	–

*N* = 779.

*p < 0.05. **p < 0.01. ***p < 0.001.

3 Mindfulness as measured in SMQ total score; 4 Cognitive fusion as measured in CFQ total score; 5 Meditation experience 6 Neuroticism; 7 Conscientiousness; 8 Agreeableness; 9 Openness to experience; 10 Extraversion.

The results for the VIF with mindfulness as the mediator (*VIF* = 1.14) and for cognitive fusion as mediator (*VIF* = 2.30) were both below 5. The results for the Breusch-Pagan test conducted on the mediation analysis with mindfulness as mediator were as follows: neuroticism (*BP* = 2.8, *p* = 0.25), agreeableness (*BP* = 35.3, *p* < 0.01), conscientiousness (*BP* = 4.6, *p* = 0.10), extraversion (*BP* = 4.3, *p* = 0.12), openness (*BP* = 6.1, *p* = 0.05). For the mediation with cognitive fusion the results were: neuroticism (*BP* = 0.83, *p* = 0.67), agreeableness (*BP* = 33.7, *p* < 0.01), conscientiousness (*BP* = 4.7, *p* = 0.09), extraversion (*BP* = 2.1, *p* = 0.36), openness (*BP* = 3.7, *p* = 0.15). Since the Breusch-Pagan test was significant for agreeableness in both models, the mediations with agreeableness as DV were additionally calculated with the use of robust Standard Errors (HC4). The significance of the effects remained unchanged in both models.

**Fig. 1:**
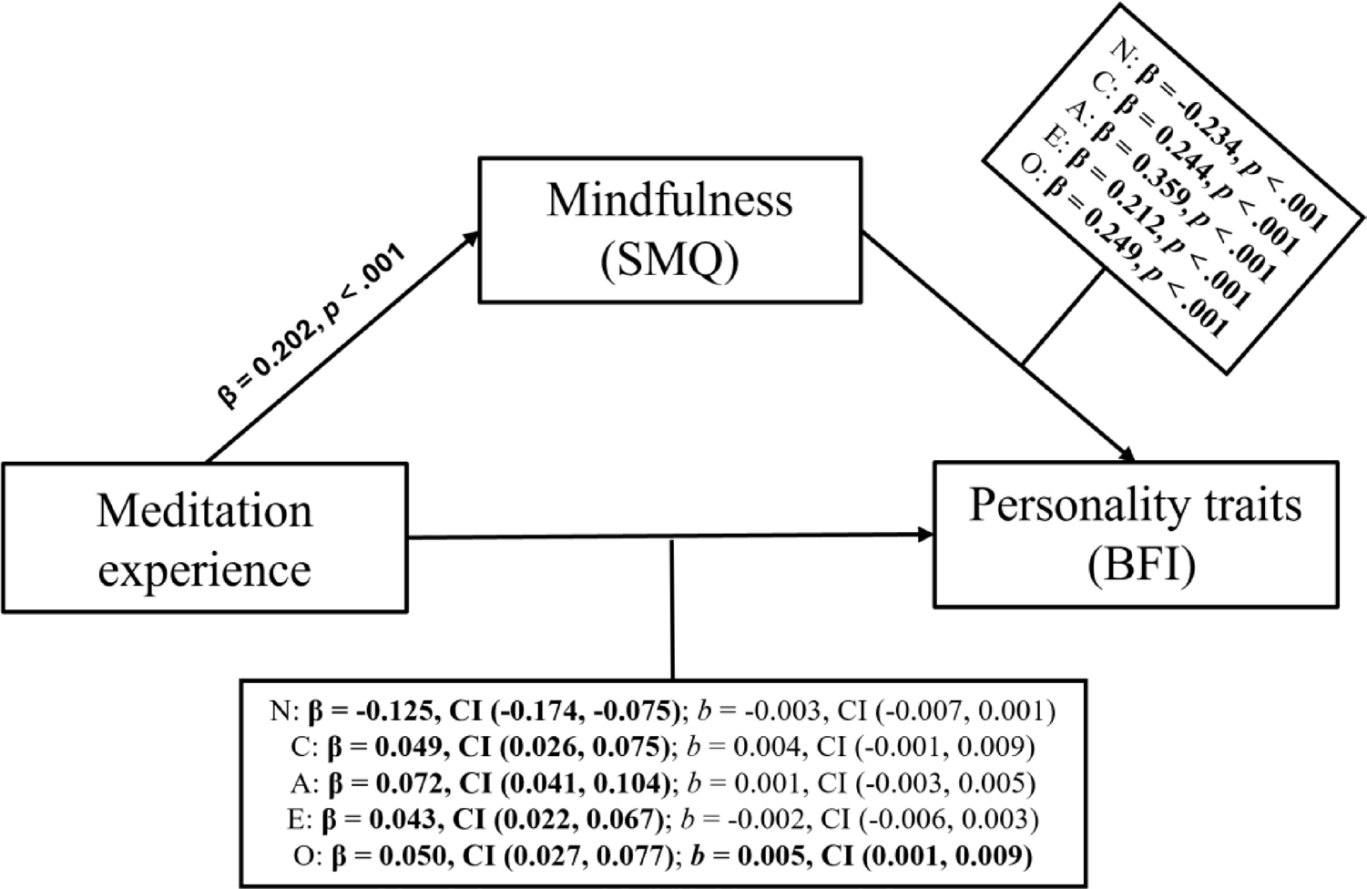
Mediation model of meditation experience, mindfulness and personality traits. *Note.* *SMQ* Southampton Mindfulness Questionnaire**,** *BFI* Big Five Inventory, *β* standardized indirect effect, *CI* 95% confidence interval, *b* direct effect, *N* Neuroticism, *C* Conscientiousness, *A* Agreeableness, *E* Extraversion, *O* Openness, bold values indicate statistical significance.

In the unadjusted model of the first hypothesis (Fig. 1) with neuroticism as the DV, the standardized indirect effect was significant (β = − 0.222; 95% CI [− 0.265, − 0.182]). Moreover, the direct effect (*b* = − 0.004; CI [− 0.007, − 0.001]) and total effect (*b* = − 0.017; CI [− 0.022, − 0.013]) were also significant. After adjusting for the covariates, the standardized indirect effect (β = − 0.125; CI [− 0.174, − 0.075]) and total effects (*b* = − 0.010; CI [− 0.015, − 0.005]) remained significant, however the direct effect became non-significant (*b* = − 0.003; CI [− 0.007, 0.001]). The adjusted model predicting neuroticism showed the following fit (*R*^*2*^ = 0.117; *F* = 34.1; *p* < 0.001).

When conscientiousness serves as the dependent variable in the unadjusted model, the standardized indirect effect (β = 0.096; CI [0.068, 0.127]), the direct effect (*b* = 0.007; CI [0.003, 0.011]), and the total effect (*b* = 0.012; CI [0.009, 0.016]) all displayed significance. When adjusting for covariates, the direct (*b* = 0.004; CI [− 0.001, 0.009]) became non-significant, while the standardized indirect effect (β = 0.049; CI [0.026, 0.075]) and total effect (*b* = 0.006; CI [0.002, 0.011]) remained significant. The adjusted model displayed the following fit (*R*^*2*^ = 0.080; *F* = 22.4; *p* < 0.001). Age was a significant covariate across all personality traits, while meditation intensity was not.

When conscientiousness was replaced with agreeableness as the dependent variable, the unadjusted model was significant in the standardized indirect effect (β = 0.137; CI [0.105, 0.171]), the direct (*b* = 0.004; CI [0.001, 0.007]) and the total effect (*b* = 0.011; CI [0.007, 0.014]). The adjusted model was significant (*R*^*2*^ = 0.084; *F* = 23.7; *p* < 0.001), with the standardized indirect effect (β = 0.072; CI [0.041, 0.104]), and the total effect (*b* = 0.004; CI [0.001, 0.008]) remaining significant, while the direct effect (*b* = 0.001; CI [− 0.003, 0.005]) did not display significance.

**Fig. 2:**
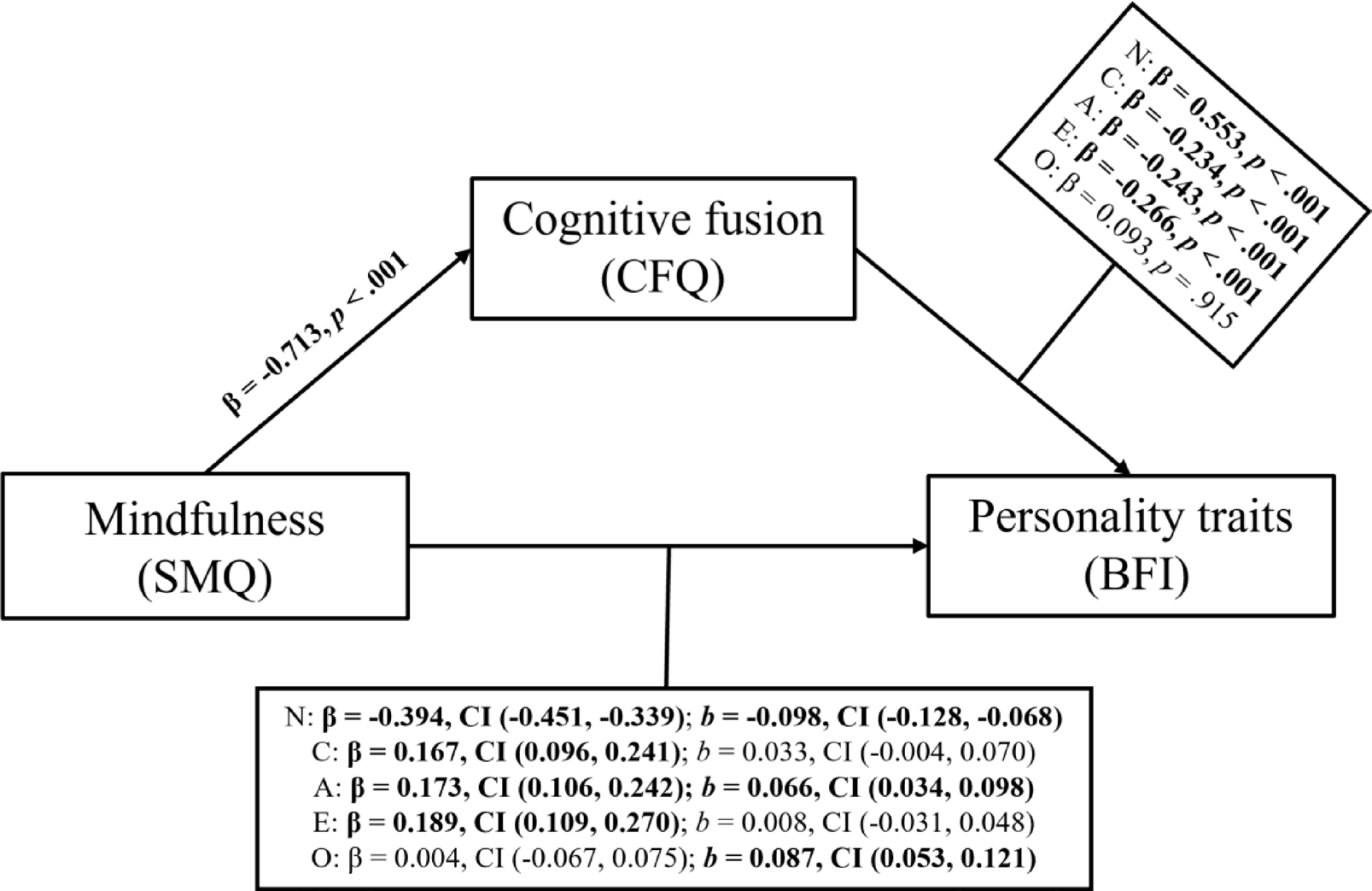
Mediation model of mindfulness, cognitive fusion and personality traits. *Note.* *SMQ* Southampton Mindfulness Questionnaire**,** *CFQ* Cognitive Fusion Questionnaire, *BFI* Big Five Inventory, *β* standardized indirect effect, *CI* 95% confidence interval, *b* direct effect, *N* Neuroticism, *C* Conscientiousness, *A* Agreeableness, *E* Extraversion, *O* Openness, bold values indicate statistical significance.

For the second hypothesis (Fig. 2), with neuroticism as the DV, the unadjusted model had a significant standardized indirect effect of (β = − 0.419; CI [− 0.48, − 0.36]). The direct effect (*b* = − 0.098; CI [− 0.13, − 0.07]) and the total effect (*b* = − 0.272; CI [− 0.29, − 0.25]) were also significant. When controlling for the two covariates, the standardized indirect effect (β = − 0.394; CI [− 0.45, − 0.34]), the direct effect (*b* = − 0.097; CI [− 0.13, − 0.07]) and the total effect (*b* = − 0.261; CI [− 0.29, − 0.24]) all remained significant. The overall model demonstrated significant results (*R*^*2*^ = 0.432; *F* = 196.2; *p* < 0.001).

When conscientiousness served as the dependent variable, the unadjusted model displayed a significant standardized indirect effect (β = 0.202; CI [0.13, 0.28]), direct effect (*b* = 0.043; CI [0.01, 0.08]) and total effect (*b* = 0.118; CI [0.09, 0.14]). Once the covariates were included, the standardized indirect effect (β = 0.167; CI [0.10, 0.24]), the direct effect (*b* = 0.033; CI [− 0.01, 0.07]) and the total effect to (*b* = 0.094; CI [0.07, 0.12]) all remained significant. The total model displayed a significant fit (*R*^*2*^ = 0.125; *F* = 37.0; *p* < 0.001). Similar to the results of the first hypothesis, meditation intensity did not show significant covariance across relevant personality traits, while age did.

Finally, when agreeableness served as the dependent variable instead of neuroticism, the unadjusted standardized indirect effect (β = 0.197; CI [0.13, 0.27]), the direct effect (*b* = 0.074; CI [0.04, 0.11]), and the total effect (*b* = 0.140; CI [0.12, 0.16]) were significant. The adjusted model had the following fit (*R*^*2*^ = 0.190; *F* = 60.4; *p* < 0.001), with the standardized indirect effect (β = 0.173; CI [0.11, 0.24]), the direct effect (*b* = 0.066; CI [0.03, 0.10]) and the total effect (*b* = 0.122; CI [0.10, 0.15]) all being statistically significant.

### Exploratory analysis

A detailed account of the mediation analyses with all five personality traits are displayed in the exploratory analysis section in Tables 3 and 4. Significance was set at α = 0.05.

**Table 3:** Results of the mediation analysis of meditation experience, mindfulness and personality traits.

	Neuroticism	Conscientiousness	Agreeableness	Openness	Extraversion
Stand. ind. effect (95% CI)	**− 0.125** **(− 0.174, − 0.075)**	**0.049** **(0.026, 0.075)**	**0.072** **(0.041, 0.104)**	**0.050** **(0.027, 0.077)**	**0.043** **(0.022, 0.067)**
Indirect effect (95% CI)	**− 0.008** **(− 0.011, − 0.004)**	0.003 (0.001, 0.004)	**0.004** **(0.002, 0.005)**	0.003 (0.001, 0.004)	**0.002** **(0.001, 0.004)**
Direct effect (95% CI)	− 0.003 (− 0.007, 0.001)	0.004 (− 0.001, 0.009)	0.001 (− 0.003, 0.005)	**0.005** **(0.001, 0.009)**	− 0.002 (− 0.006, 0.003)
Total effect (95% CI)	**− 0.011** **(− 0.015, − 0.005)**	**0.007** **(0.002, 0.011)**	**0.005** **(0.001, 0.008)**	**0.007** **(0.003, 0.011)**	0.001 (− 0.004, 0.006)

Bold values indicate significance.

**Table 4:** Results of the mediation analysis of mindfulness, cognitive fusion and personality traits.

	Neuroticism	Conscientiousness	Agreeableness	Openness	Extraversion
Stand. ind. effect (95% CI)	**− 0.394** **(− 0.451, − 0.339)**	**0.167** **(0.096, 0.241)**	**0.173** **(0.106, 0.242)**	0.004 (− 0.067, 0.075)	**0.189** **(0.109, 0.270)**
Indirect effect (95% CI)	**− 0.164** **(− 0.190, − 0.140)**	**0.062** **(0.035, 0.089)**	**0.058** **(0.034, 0.082)**	0.001 (− 0.023, 0.025)	**0.072** **(0.041, 0.103)**
Direct effect (95% CI)	**− 0.098** **(− 0.128, − 0.068)**	0.033 (− 0.004, 0.070)	**0.066** **(0.034, 0.098)**	**0.087** **(0.053, 0.121)**	0.008 (− 0.031, 0.048)
Total effect (95% CI)	**− 0.262** **(− 0.285, − 0.238)**	**0.095** **(0.068, 0.121)**	**0.124** **(0.101, 0.147)**	**0.088** **(0.064, 0.112)**	**0.080** **(0.052, 0.108)**

Bold values indicate significance.

## Discussion

The present study investigated two research questions. Firstly, we tested whether mindfulness mediates the relationship between meditation experience and personality. This analysis aimed to explore whether mindfulness meditation may potentially shape personality traits according to the Big Five model, as suggested in previous research^16,24,25^.

Our findings show that mindfulness significantly mediates the relationship between meditation experience and personality across all five personality traits. More specifically, individuals with greater meditation experience report significantly higher levels of mindfulness, which in turn is associated with lower neuroticism and higher levels of conscientiousness, agreeableness, extraversion, and openness. These results align with existing literature and our predictions^10,12,26^, and provides support for the hypothesis that mindfulness meditation contributes to personality change. The strongest standardized indirect effect was observed for neuroticism, a trait linked to affective lability, anxiety and vulnerability to stress^13^. Neuroticism is a well-established risk factor for suffering from anxiety and depressive disorders, as well as general psychological distress^13^. Even modest reductions at the trait level may lead to clinically relevant protective effects^18,43,44^. On an individual level, consistent meditative practice over time may yield cumulative, incremental changes in aspects of neuroticism such as emotional instability, leading to increased resilience and substantial improvements in psychological well-being^45,46^. MM may therefore be especially well-suited to individuals with high levels of neuroticism, offering a promising opportunity for the development of more individualized mindfulness-based treatments. Prior research has suggested that personality traits, such as neuroticism, may influence how individuals respond to and benefit from mindfulness-based interventions^14^, and evidence from student samples shows that those high in neuroticism experience greater benefits^15^. Tailoring such treatments to specific personality traits could enhance their effectiveness by targeting those who are most likely to benefit.

Beyond neuroticism, mindfulness also displayed positive associations with conscientiousness and agreeableness, traits characterized by discipline, reliability, empathy and prosocial behavior^13^. Notably, research has shown that conscientiousness and agreeableness increase with age, while neuroticism tends to decrease from adolescence to adulthood^33^. These findings align with the association between mindfulness and personality^10,12^. Interestingly, this parallel suggests that meditative practice may promote a personality pattern similar to that of individuals with greater life experience. This interpretation is further supported by our own findings, where age emerged as a significant covariate across all our mediation models. Including age as a covariate in future studies on this topic is integral to untangle age-related personality trait changes from the effects of mindfulness practice itself. In contrast, meditation intensity, measured in weekly hours of meditative practice, did not function as a significant covariate in any of our models. This suggests that cumulative meditation experience may be more relevant than current practice frequency, when assessing the impact of mindfulness on personality traits.

It is important to acknowledge that personality traits are relatively stable constructs, influenced by genetic, developmental and environmental factors^47^. As such, it is unsurprising that the effect sizes observed in our study were relatively small for a single factor such as meditative practice. Nonetheless, multiple studies have demonstrated that a modifiable construct like mindfulness displays significant associations with personality traits^16,24,25^, suggesting that mindfulness meditation could promote lasting improvements in mental wellbeing. When considered across a large population, even modest reductions in neuroticism could result in fewer individuals crossing the threshold into clinical anxiety or depression. Still, to draw more definite conclusions, the base of evidence must be strengthened through large, longitudinal studies. Our cross-sectional findings should be regarded as valuable, but preliminary groundwork, enabling more rigorously designed research. At the same time, advancing this area of research requires clarifying the psychological mechanisms through which mindfulness exerts its influence on personality.

In this context, our second hypothesis aimed to improve our understanding of how mindfulness affects personality, by exploring the mediating role of cognitive fusion. Our results show that cognitive fusion is a significant mediator for each of the three relevant personality traits, confirming our second hypothesis on a diverse sample of meditators. The mediation between mindfulness and neuroticism is distinguished by its notably larger total effect compared to other personality traits. 63% of this total effect is mediated through cognitive fusion, highlighting its central role in this relationship. This suggests that reductions in cognitive fusion account for a substantial proportion of the overall influence mindfulness exerts on this trait. These results indicate that the observed association between mindfulness and neuroticism might be primarily linked through changes in cognitive flexibility. This may correspond to decreases in the experience of emotional distress and negative affect since both constructs are closely associated with neuroticism^48^.

Our results therefore align with previous research highlighting the association between cognitive fusion, within the framework of psychological flexibility, and reduced negative and depressive symptoms in individuals with schizophrenia spectrum disorders (SSD)^49^. Further underscoring the relevant role of cognitive fusion in mental health outcomes, recent studies identified cognitive fusion as a mediator in the relationship between mindfulness and reductions in symptom severity among individuals with SSD^50^ and in the association between mindfulness and decreased negative affect^29^. This pattern is also reflected in the practice of Acceptance and Commitment Therapy (ACT)^51^. As pointed out by Gillanders et al.^27^, ACT targets present-moment awareness as a preliminary step to initiate cognitive defusion, aiming to increase psychological well-being in the short term. By showing that mindfulness also influences the more enduring concept of personality, specifically neuroticism, through increases in cognitive flexibility, our results suggest that the impact of mindfulness may extend beyond reductions in negative affect and symptom severity, potentially leading to more long-term improvements in psychological well-being. These findings highlight the therapeutic potential of mindfulness-based interventions, particularly those targeting cognitive fusion, such as ACT and other third-wave CBT approaches, to promote lasting mental health improvements. The implications may extend beyond clinical settings, supporting the value of meditative practice in promoting psychological resilience.

Apart from its influence on neuroticism, the indirect effect was also significant for agreeableness and conscientiousness. Compared to neuroticism, the magnitude of the standardized indirect effect was more moderate. Nonetheless, cognitive fusion also accounted for a large part of the effect of mindfulness on these traits, with 68% for conscientiousness and 47% for agreeableness being mediated through the indirect pathway. Interestingly, the results for extraversion were significant and comparable in magnitude to conscientiousness and agreeableness. To our surprise, cognitive fusion fully mediated the association between mindfulness and extraversion, as the direct effect became non-significant once the mediator was added, accounting for approximately 90% of the total effect. The non-significant direct effect aligns with extensive evidence^10,12^, outlining that extraversion is typically weakly associated with mindfulness. This full mediation is an important new finding since it reveals a previously unrecognized indirect pathway through which mindfulness does, in fact, seem to influence extraversion. Similarly, with the addition of covariates, cognitive fusion fully mediated the relationship between mindfulness and conscientiousness. This underscores the importance of accounting for changes in psychological flexibility, when examining how mindfulness affects personality traits. As expected, the results concerning openness to experience were insignificant.

Notably, as in the first hypothesis, age was a significant covariate across all models. Beyond its discussed association with mindfulness and personality, age was found to significantly influence levels of cognitive fusion as well^52^, making its continued significance as a covariate unsurprising. Meditation intensity did not display statistical significance.

Cognitive flexibility seems to be a key mechanism through which mindfulness affects several aspects of personality. It appears to have an especially strong influence on neuroticism, suggesting that changes in cognitive fusion may significantly contribute to the association between mindfulness and mental well-being.

### Strengths and limitations

A notable strength of this study is its large sample size of 779 participants, which enhances the statistical power and reliability of our results. Additionally, collecting data on a diverse sample of regular meditators with varied backgrounds provides a comprehensive context for examining mindfulness, cognitive fusion and personality traits.

We also acknowledge that this study has several limitations. The most important one is its cross-sectional design, which prevents conclusions about causality. Therefore, our mediation models serve as atemporal models of mediation^53^. The direction of relationships between mindfulness, cognitive fusion and personality, as well as meditation experience, mindfulness and personality cannot be established, and further longitudinal trials are needed to confirm the causal pathways behind our suggested model.

An additional contextual limitation concerns the timing of data collection (June 2020 to August 2021), which coincided with heightened COVID-19 infection rates and widespread public health restrictions. Pandemic-related conditions, such as elevated distress levels, limited mobility and lifestyle restrictions may have influenced responses, particularly levels of cognitive fusion. This context should be considered when interpreting the results.

The data primarily comes from meditators in Western countries, limiting the generalizability of these findings. Although the survey was available in English and distributed to meditation centers worldwide, recruitment was limited by access to those centers and their internet presence. Although such limitations are inherent to international online surveys, they limit the generalizability of our results.

### Future directions

The results for both of our hypotheses offer a promising foundation for future explorations. Specifically, both research questions could be combined into one longitudinal study over an extended period of time, researching the change of personality traits in response to mindfulness meditation, and exploring whether changes in cognitive fusion mediate this influence. Replication using longitudinal designs are integral to confirm and extend the current results, address the limitations of our cross-sectional design, and clarify the causal direction of the relationship between the variables.

Additionally, the current findings highlight a promising direction for developing more individualized mindfulness-based interventions by tailoring them to personality traits such as neuroticism, thereby enhancing their effectiveness by targeting those likely to benefit the most.

### Conclusion

This article explored the relationship between mindfulness and personality, focusing on two research questions. First, we investigated whether mindfulness mediates the association between meditation experience and personality traits. Our findings demonstrate that mindfulness significantly mediates this relationship across all five traits of the Big Five model, supporting existing literature. Most notably, the strongest mediated effect was observed for neuroticism, a trait linked to emotional instability and psychological distress. This suggests that mindfulness meditation may contribute to changes in personality, particularly by reducing neuroticism, promoting lasting improvements in mental wellbeing.

Second, to elucidate how mindfulness influences personality traits, our findings highlight the central role of cognitive fusion as a mediator. Our analysis suggests that changes in cognitive flexibility account for a substantial proportion of the association between mindfulness and neuroticism and, to a lesser extent, for conscientiousness and agreeableness. Additionally, we identified a previously unrecognized indirect pathway, as cognitive fusion fully mediates the effect of mindfulness on extraversion, a personality trait consistently found to be minimally associated with mindfulness. Together, these results suggest that mindfulness meditation may have a transformative influence on personality structure, with increased cognitive flexibility emerging as a key psychological mechanism underlying these changes.

## Data Availability

The data and code supporting the findings of this study are openly available on the Open Science Framework (OSF). The repository includes the processed data and the analysis script. These materials can be accessed through the following link: https://osf.io/spv48/ This link directly leads to the download of our dataset: https://osf.io/download/hbv5n/
